# Performance and usefulness of a novel automated immunoassay HISCL SARS-CoV-2 Antigen assay kit for the diagnosis of COVID-19

**DOI:** 10.1038/s41598-021-02636-x

**Published:** 2021-12-01

**Authors:** Kaori Saito, Tomohiko Ai, Akinori Kawai, Jun Matsui, Yoshiyuki Fukushima, Norihiro Kikukawa, Takuya Kyoutou, Masayoshi Chonan, Takeaki Kawakami, Yoshie Hosaka, Shigeki Misawa, Haruhi Takagi, Yasushi Matsushita, Makoto Hiki, Atsushi Okuzawa, Satoshi Hori, Toshio Naito, Takashi Miida, Kazuhisa Takahashi, Yoko Tabe

**Affiliations:** 1 Department of Clinical Laboratory Medicine, Juntendo University Graduate School of Medicine, 2-1-1, Hongo, Bunkyo-ku, Tokyo, 113-8421 Japan; 2 Clinical Innovation, Sysmex Corporation, Kobe, Japan; 3 LS Medical Affairs, Sysmex Corporation, Kobe, Japan; 4 Engineering 1, Sysmex Corporation, Kobe, Japan; 5 Department of Clinical Laboratory, Juntendo University Hospital, Tokyo, Japan; 6 Department of Respiratory Medicine, Juntendo University Graduate School of Medicine, Tokyo, Japan; 7 Department of Internal Medicine and Rheumatology, Juntendo University Graduate School of Medicine, Tokyo, Japan; 8 Emergency and Disaster Medicine, Juntendo University Faculty of Medicine, Tokyo, Japan; 9 Department of Cardiovascular Biology and Medicine, Juntendo University Faculty of Medicine, Tokyo, Japan; 10 Department of Coloproctological Surgery, Juntendo University Graduate School of Medicine, Tokyo, Japan; 11 Department of Infection Control Science, Juntendo University Graduate School of Medicine, Tokyo, Japan; 12 Department of General Medicine, Juntendo University Graduate School of Medicine, Tokyo, Japan; 13 Department of Next Generation Hematology Laboratory Medicine, Juntendo University Graduate School of Medicine, Tokyo, Japan

**Keywords:** Virology, SARS-CoV-2

## Abstract

Here, we aimed to evaluate the clinical performance of a novel automated immunoassay HISCL SARS-CoV-2 Antigen assay kit designed to detect the nucleocapsid (N) protein of severe acute respiratory syndrome coronavirus 2 (SARS-CoV-2). This kit comprises automated chemiluminescence detection systems. Western blot analysis confirmed that anti-SARS-CoV antibodies detected SARS-CoV-2N proteins. The best cut-off index was determined, and clinical performance was tested using 115 nasopharyngeal swab samples obtained from 46 patients with coronavirus disease 2019 (COVID-19) and 69 individuals who tested negative for COVID-19 through reverse transcription quantitative polymerase chain reaction (RT-qPCR). The HISCL Antigen assay kit showed a sensitivity of 95.4% and 16.6% in samples with copy numbers > 100 and < 99, respectively. The kit did not cross-react with human coronaviruses causing seasonal common cold and influenza, and none of the 69 individuals without COVID-19 were diagnosed with positive results. Importantly, 81.8% of the samples with low virus load (< 50 copy numbers) were diagnosed as negative. Thus, using HISCL antigen assay kits may reduce overdiagnosis compared with RT-qPCR tests. The rapid and high-throughput HISCL SARS-CoV-2 Antigen assay kit developed here proved suitable for screening infectious COVID-19 and may help control the pandemic.

## Introduction

The coronavirus disease-2019 (COVID-19) pandemic originating from Wuhan, China has caused chaos and health and economic crises across the world^1–7^ despite the cases of infection and death being far fewer than those of the 1918 influenza pandemic^8^. The World Health Organization declared COVID-19 a pandemic in March 2020.

Since severe acute respiratory syndrome coronavirus 2 (SARS-CoV-2) is contagious in humans, it is important to determine the infection status of individuals accurately^9^. During the early phase of the pandemic, a reverse transcriptase quantitative-polymerase chain reaction (RT-qPCR)-based test was considered a gold standard. However, a meta-analysis of 51 studies reported that the overall sensitivity of PCR tests was 89.1% and the specificity was 98.9%, even though the true definition of COVID-19 remains ambiguous^10^. In addition, results can vary because the accuracy of PCR tests can be significantly affected by various factors, such as primer design and sample collection techniques, reagents, and laboratory equipment. Furthermore, patients can test positive for COVID-19 by PCR tests during post-infectious periods^11^ as the cut-off can be changed by altering the cycle threshold (Ct) values. For example, low, clinically insignificant amounts of viral RNAs can still be detected when the Ct is set to more than 35, suggesting a positive result. This indicates that the relationship between Ct values and infectious status is debatable^12–14^ since non-infectious COVID-19 individuals can be labeled as positive due to overdiagnosis by RT-qPCR tests^15–17^. This issue raises serious concerns, not only in terms of medical decisions but also with regard to the global economy and the protection of human rights^18–21^.

Recently, SARS-CoV-2 Antigen detection assays were developed as potential alternative tests to RT-qPCR to identify infectious patients, as stated by the U.S. Food and Drug Administration (https://www.fda.gov/consumers/consumer-updates/coronavirus-disease-2019-testing-basics). In the early phase of the COVID-19 pandemic, Ag Respi-Strip, an immunochromatographic assay, was developed (Coris BioConcept, Gembloux, Belgium) in which monoclonal antibodies against the nucleocapsid (N) protein of SARS-CoV were used. The LHUB-ULB SARS-CoV-2 working diagnostic group reported that the COVID-19 Ag Respi-Strip assay showed an overall sensitivity and specificity of 57.6% and 99.5%, respectively^22^. In June 2020, LUMIPULSE G1200 using a chemiluminescence enzyme immunoassay became available (Fujirebio, Tokyo, Japan), and its overall sensitivity and specificity in Japanese patients was 55.2% and 99.6%, respectively^23^.

We developed an automated antigen detection system, which we termed as the HISCL SARS-CoV-2 Antigen assay kit that can detect the N protein of SARS-CoV-2 using enzyme-linked immunosorbent assay (ELISA). This automated test can process 200 samples per hour, making it suitable for mass screening. In this study, we examined the feasibility and accuracy of the HISCL SARS-CoV-2 Antigen assay kit for screening COVID-19 patients in Japan.

## Results

### Western blot

**Figure 1:**
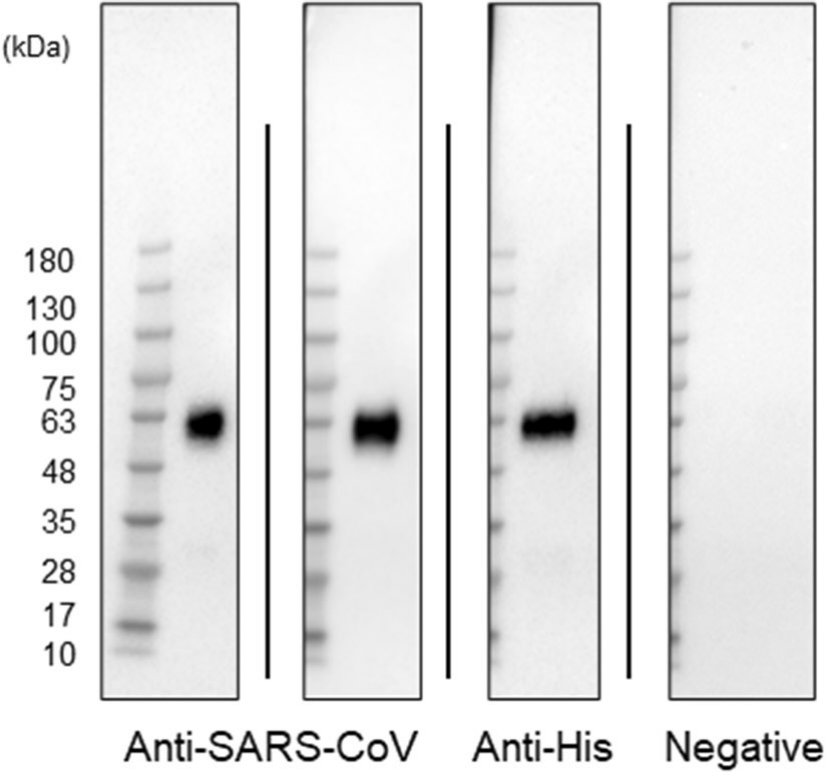
Western blot analysis. Anti-SARS-CoV antibodies (left two strips) and anti-His antibodies (third strip form the left) reacting with SARS-CoV-2 antigens. The far-right strip shows negative control (without antigens). All strips were cropped from a membrane shown in Supplemental Figure 1. SARS-CoV-2: severe acute respiratory syndrome coronavirus 2; His: histidine.

To examine whether the previously produced anti-SARS-CoV antibodies (ab clone# 2–3 and ab clone# 2–12)^24^ can detect SARS-CoV-2, western blots were performed using His-tagged recombinant N proteins of SARS-CoV-2 (ACRO Biosystems, Newark, DE). Figure 1 shows that the preanti-SARS-CoV antibodies (left two strips: ab clone# 2–3 and ab clone# 2–12) and anti-His antibody (the third strip from the left) detected the SARS-CoV-2 antigens but not negative control (the far-right strip). The original stained membrane is shown in Supplemental Figure 1.

### Determination of the association between Ct values and viral loads

**Figure 2:**
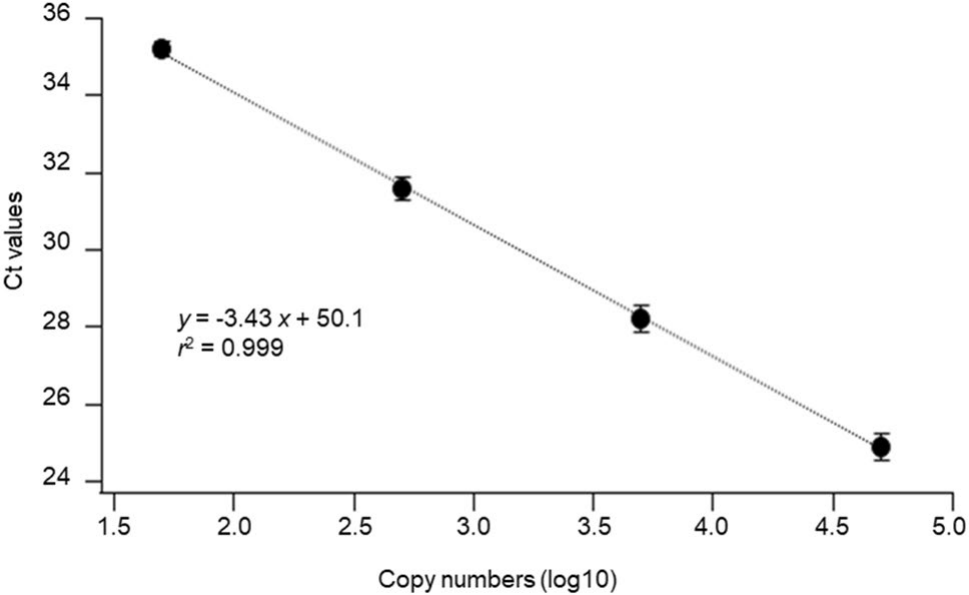
Standard curve for relations between Ct values and copy numbers. Error bars indicate SD. *Ct* cycle threshold, *SD* standard deviation.

Since RT-PCR results can be affected by different laboratory settings^25^, relationship between Ct values by a RT-qPCR method^26^ and viral copy numbers was determined in our laboratory. SARS-CoV-2 Positive Control RNA (1 × 10^5^ copies/µL; JP-NN2-PC, Nihon Gene Research Laboratories) was sequentially diluted (50 to 5,000 copies/sample) and subjected to RT-qPCR. Figure 2 shows the standard curve of Ct values as a function of copy numbers using four RNA samples for each copy number. The plots were fitted with linear regression, yielding the slope of − 3.43 and the intercept of 50.1 (*r*^2^ = 0.999).

### HISCL SARS-CoV-2 Antigen assay kit

As described in the Methods section, recombinant SARS-CoV-2 proteins (ACRO Biosystems, Newark, DE) were incubated with biotinylated SARS-CoV-2 antibodies (R1: ab 2–3) and alkaline phosphatase (ALP)-bound SARS-CoV-2 antibodies (R3: ab 2–12). After washing out the unbound proteins, chemiluminescent substrates (CDP-Star, C0712, Sigma-Aldrich, St. Louis, MO, USA) were added, and fluorescence signals were measured using the photo counter of HISCL-800 (Sysmex, Kobe, Japan). The level of SARS-CoV-2 antigens was indicated as the cut-off index (COI), calculated by the difference in the luminescence intensities in the buffers with and without the SARS-CoV-2 antigens. The COI was defined as:

Cut-off index (C.O.I.) = (light intensity in a sample—light intensity with the negative control) / (light intensity at a calculated cut-off value—light intensity with the negative control).

### Reproducibility

To check the reproducibility of the HISCL SARS-CoV-2 Antigen assay kit, we measured one sample containing recombinant SARS-CoV-2 antigens (109.5 ± 36.5 pg/mL, ACRO Biosystems, Newark, DE) (positive control sample) and one sample containing only buffers (negative control sample). The tests were repeated ten times for each sample and all experiments were conducted in triplicate. Table 1 shows the COI values for both samples. The negative control samples showed 100% agreement with COI of zero and the positive control samples showed the average COI of 27.7 to 28.8 with a coefficient of variation (CV) percentage of 1.3–2.5.

**Table 1:** Within-run reproducibility. The COI values of samples without or with SARS-CoV-2 antigens were measured a total of 30 times (three sets of 10 consecutive measurements).

Control panels	COI		
**Negative control**			
1	0	0	0
2	0	0	0
3	0	0	0
4	0	0	0
5	0	0	0
6	0	0	0
7	0	0	0
8	0	0	0
9	0	0	0
10	0	0	0
**Positive control**			
1	28.4	27.9	29
2	29	27.3	29
3	29	26.3	27.3
4	28.4	27.9	27.9
5	29	27.3	27.9
6	28.4	27.9	27.9
7	28.4	27.9	28.4
8	29	28.4	28.4
9	27.9	29	27.9
10	29	27.3	27.9
Average	28.7	27.7	28.2
SD	0.4	0.7	0.5
CV (%)	1.3	2.5	1.8

*COI* cutoff index, *SD* standard deviation, *CV* coefficient of variation.

To check reproducibility between measurements in five different days, we performed assays using the negative and positive control samples twice daily for 5 days. Table 2 shows that the COIs of the negative and positive control samples were consistent (CV% < 10).

**Table 2:** Between-run reproducibility.

COI	Run1			Run2		
	1	2	Mean	1	2	Mean
**Negative control**						
Day1	0	0	0	0	0	0
Day2	0.1	0.1	0.1	0	0.1	0
Day3	0	0	0	0	0	0
Day4	0	0	0	0	0	0
Day5	0	0	0	0	0	0
**Positive control**						
Day1	26.8	27.3	27	26.7	27	26.8
Day2	27.5	28.1	27.8	28	27.2	27.6
Day3	28.4	28.5	28.4	29	27.7	28.3
Day4	28.2	28	28.1	28.6	28.4	28.5
Day5	30.7	30.6	30.6	30.8	29.8	30.3
		Average	28.4		Ave	28.3
		SD	1.3		SD	1.3
		CV (%)	4.7		CV (%)	4.6

*COI* cut-off index, *SD* standard deviation, *CV* coefficient of variation.

### Cross-reaction

To check for specificity, various corona and influenza viral antigens were used to measure COIs in the HISCL assay. Table 3 summarizes the COI measurements. The COI showed high values for SARS-CoV at a concentration of 0.25 ng/mL and positive values for MERS-CoV at a low concentration. In contrast, the COI values stayed in the negative range for other coronaviruses and influenza viruses, indicating that the HISCL SARS-CoV-2 Antigen assay kit does not react with seasonal common cold and flu viruses.

**Table 3:** Cut-off index (COI) values in negative controls.

Panels	Concentration (ng/mL)								
	0	0.25	0.5	0.75	1	25	50	75	100
MERS-CoV	0	1.1	2.3	3.3	4.6	11.9	23.6	34.3	46.2
HCoV-229E	0	–	–	–	–	0	0	0	0
HCoV-OC43	0	–	–	–	–	0	0.1	0.2	0.2
HCoV-NL63	0	–	–	–	–	0	0	0	0.1
HCoV-HKU1	0	–	–	–	–	0	0.1	0	0.1
SARS-CoV	0	26	53	75.2	109.8	2938	5727.1	8127.1	10,682.1
Influenza H1N1	0	–	–	–	–	0.1	0	0	0
Influenza H3N2	0	–	-	–	–	0	0	0	0
Influenza B	0	–	–	–	–	0	0	0	0

*HCoV* human coronavirus, *MERS-CoV* Middle East respiratory syndrome-related coronavirus, *SARS-CoV* severe acute respiratory syndrome coronavirus, *SARS-CoV-2* severe acute respiratory syndrome coronavirus 2.

### Determination of the cut-off value

To optimize the cut-off antigen level for accurate diagnoses, we first determined the relationship between COI and viral loads using a total of 84 nasopharyngeal swabs collected from 17 patients at Kobe City Medical Center General Hospital and 67 commercially available samples (Cantor Bioconnect, Santee, CA, USA): 30 positive samples and 54 negative samples.

**Figure 3:**
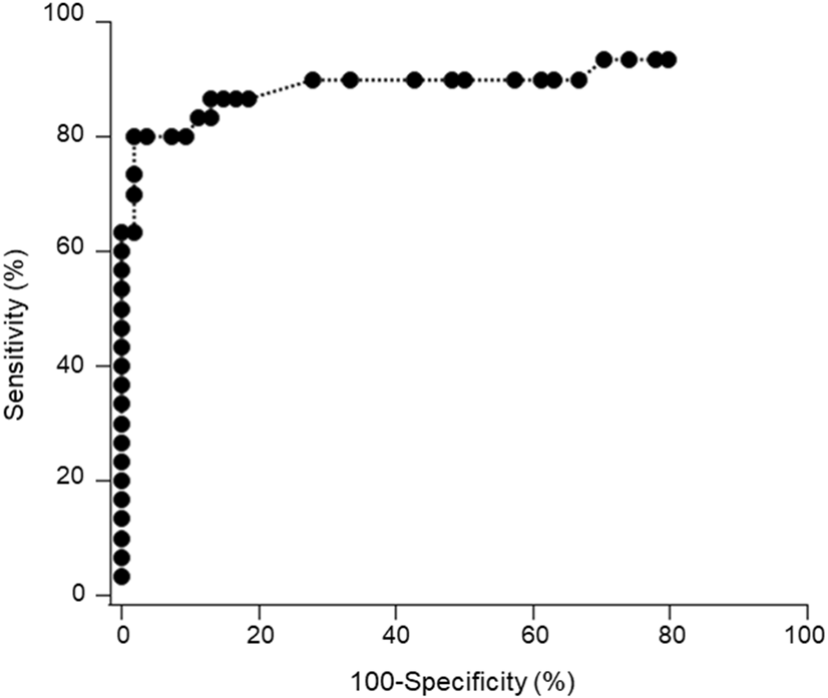
Receiver operating characteristic (ROC) curve analysis. An ROC curve was drawn using human samples with and without SARS-CoV-2.

Chemiluminescence counts were measured by HISCL SARS-CoV-2 Antigen assay kit using these samples and recombinant SARS-CoV-2 proteins (0, 20, and 100 pg/mL). Viral loads (copy numbers) were calculated based on the relationships between Ct values and RNA content (Supplemental Table 1). Sensitivity and specificity were calculated at different cut-off values depending upon the RT-qPCR results (i.e., copy number 0 as negative). In determination of the threshold, we put priority on obtaining best specificity according to the Antigen Testing Algorithm by the Center for Disease Control (https://www.cdc.gov/coronavirus/2019-ncov/lab/resources/Antigen_Testing_Algorithm_2020-12-14_v03_NO_DRAFT_SPW_508.pdf). When the cut-off for the antigen level was set to 3.65 pg/mL, sensitivity of 80% and specificity of 98.2% were obtained (Table 4). Thus, chemiluminescence count using 3.65 pg/mL antigen was set to a COI value of 1.0 in the HISCL SARS-CoV-2 Antigen assay kit. Figure 3 shows that the ROC analysis yielded an AUC value of 0.8988 ± 0.0464 (95% confidence interval, 0.808–0.990, *p* < 0.0001).

**Table 4:** Sensitivity and specificity according to various cut-off values.

Cut-off value SARS-CoV-2 Ag (pg/mL)	Sensitivity (%)	Specificity (%)
1.65	86.7	81.5
2.6	80	90.7
3.65	80	98.2
4.45	73.3	98.2
6	63.3	98.2

*SARS-CoV-2* severe acute respiratory syndrome coronavirus 2.

Table 5 summarizes the concordance between the two tests in three viral load ranges using the cut-off value in validation samples.

**Table 5:** Relationship between the SARS-CoV-2 viral loads and COI in the assay kit using commercially available samples.

RT-qPCR	SARS-CoV-2 Ag test		Total
(copies/test)	Positive	Negative	
1–50	1	4	5
51–99	4	1	5
> 100	19	1	20

*COI* cut-off-index, *SARS-CoV-2* severe acute respiratory syndrome coronavirus 2, *RT-qPCR* reverse transcription quantitative polymerase chain reaction, *Ag* antigen.

### Clinical performance

**Figure 4:**
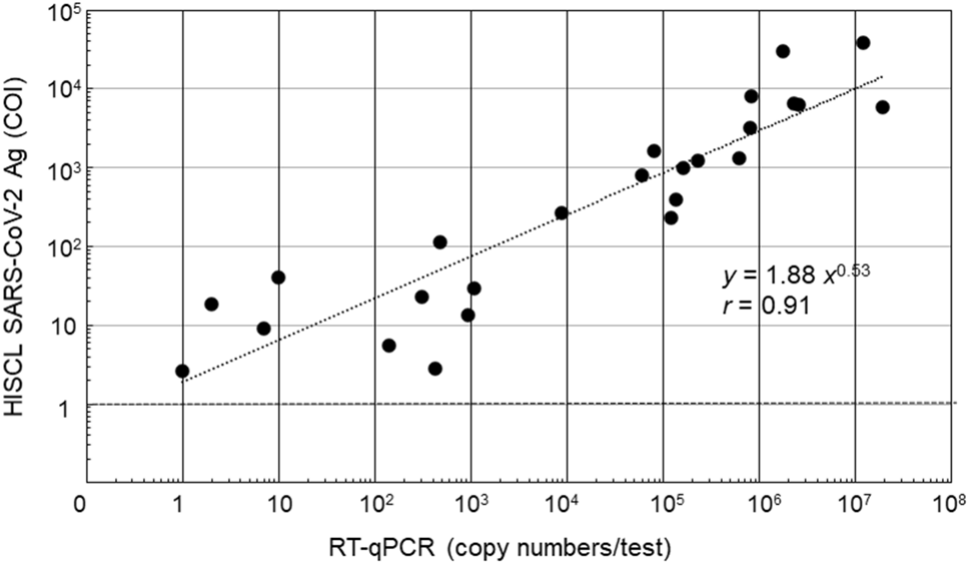
A scatter plot of HISCL SARS-CoV-2 Antigen (COI) and copy numbers measured by RT-qPCR tests. Data are plotted as logarithmic scales and the plots were fitted with a power approximation.

To examine the clinical performance, a total of 115 nasopharyngeal swab samples obtained from 115 patients at Juntendo University Hospital were subjected to the RT-qPCR and the HISCL SARS-CoV-2 Antigen assay kit. The RT-PCR test showed 46 positive and 69 negative results. The SARS-CoV-2 viral loads determined by RT-qPCR and the antigen levels detected by HISCL SARS-CoV-2 Antigen assay kit for each patient are listed in Supplemental Table 2. Figure 4 shows a dot plot of the positive COI values (> 1.0) against the viral copy numbers. The positive COI values obtained within 10 days from the onset for mild cases (n = 16) and moderate–critical cases (n = 5) had a mean [interquartile range] of 330.5 [27.6; 3849.3] and 233.9 [5.5; 1241.3], respectively (p = 0.860). Table 6 summarizes the concordance between the two tests in the two viral load ranges. The antigen tests showed 95.4% positive results in samples whose viral copy numbers were higher than 100. In samples whose copy numbers were lower than 99, the antigen test showed negative results in 20 samples and positive results in four samples.

**Table 6:** Relationship between the SARS-CoV-2 viral loads and COI by the HISCL SARS-CoV-2 Antigen assay kit.

RT-qPCR	SARS-CoV-2 Ag test		Total
(copies/test)	Positive	Negative	
1–99	4	20	24
> 100	21	1	22

*SARS-CoV-2* severe acute respiratory syndrome coronavirus 2, *RT-qPCR* reverse transcription quantitative polymerase chain reaction, *Ag* antigen.

## Discussion

In this study, we evaluated the performance of HISCL SARS-CoV-2 Antigen assay kit using chemiluminescent enzyme immunoassay in comparison with RT-qPCR test results. This test was approved by the Pharmaceuticals and Medical Device Agency of Japan in November 2020, and it is currently commercially available (Sysmex, Kobe, Japan). This automated test can process 200 samples per hour, and the run time is only 17 min. Therefore, it can be used as a high-throughput screening test.

Since the early phase of this pandemic, RT-qPCR tests have been used for diagnoses, epidemiological studies, and political decision making^27–29^. Severe cases with high infectivity usually show high viral loads (i.e., low Ct values) and vice versa^30^. However, the interpretation of positive results needs extra caution because Ct values can vary due to differences in PCR conditions^31^. Importantly, infectivity cannot be determined solely based on RT-qPCR results^11^.

To examine the correlation between RT-qPCR and our new antigen detection assay, we used SARS-CoV-2 RNAs as a standard. Our assay showed 95.4% concordance in the samples with a COI > 100, and the concordance decreased to 16.7% in samples with a COI < 99. LUMIPULSE assay (Fujirebio, Tokyo, Japan) showed 100% concordance in samples with > 100 copies^23^. This may be due to the small sample numbers used in both the studies, but it may also be attributed to the difference in the standard RNA (AccuPlex SARS-CoV-2 Reference Material Kit; SeraCare, Milford, MA, USA) and RT-qPCR methods (QuantiTect Probe RT-PCR Kit and Applied Biosystems 7500 Fast Real-Time PCR System versus StepOnePlus Real-Time PCR System).

Nonetheless, this difference should not be problematic in clinical practice since the viral loads in infectious patients were usually much higher than copy numbers of 100. For example, Yu et al. reported that copy number of SARS-CoV-2 in sputum samples in early-phase infection was 46.800 ± 17.272, and 1.252 ± 1.027 in the recovery phase^32^. Wölfel et al*.* reported that the pharyngeal virus shedding was high during the first week of symptoms, with a peak at 7.11 × 10^8^ RNA copies per throat swab on day 4^33^. Conversely, many studies also reported that there was no apparent correlation between viral loads (copy numbers) and severity of disease or mortality in patients with COVID-19 disease^34,35^. Indeed, the viral loads in our study were much lower than those in the previously reported cases, and mild cases showed similar COIs compared to moderate–critical cases (Supplemental Table 2). These data, including our data, indicate that infectivity and viral loads are not proportional. Thus, we propose this antigen test is considered as a useful screening test. However, diagnosis and clinical decisions should not be made upon any single test. Therapeutic strategies should be determined based on systemic physiological parameters, such as respiratory rate, blood oxygenation, heart rate, and blood pressure, along with the screening test.

There were a few limitations of this study: (1) this study was performed only in Japan with a relatively small number of patients; (2) we could not test the assay for mutated pathogens that might not be bound by our antibody; (3) cross-reactivity with unknown pathogens could not be excluded. However, diagnoses of COVID-19 may not benefit clinical practice since no proven remedies are available to date; and (4) we could not examine chronological changes of the antigen test results.

In conclusion, considering the current chaotic situation across the world due to COVID-19, which is in part caused by detecting non-infectious patients with extremely low viral load as positive cases by RT-qPCR, our antigen detection assay may be more suitable than RT-qPCR for mass screening as it would help to moderate COVID-19 overdiagnoses. Further validation of the kit with a larger number of samples and SARS-CoV-2 variants is warranted.

## Methods

### Patient samples

This research related to human use complied with all the relevant national regulations, institutional policies and was conducted in accordance the tenets of the Helsinki Declaration. It has been approved by the authors’ institutional review board at Juntendo University Hospital, Tokyo, Japan (IRB #20-036, Chair: Atsushi Okuzawa, MD) and Kobe City Medical Center General Hospital, Hyogo, Japan (IRB #zn200615, Chair: Yasushi Naito, MD). This study used an opt-out consent method. The Institutional Review Board of Juntendo University and the Institutional Review Board of Kobe City Medical Center General Hospital waived the need for informed consent. Therefore, written informed consent was not required.

Classification of disease severity was determined according to the Clinical Spectrum of SARS-CoV-2 Infection (https://www.covid19treatmentguidelines.nih.gov/overview/clinical-spectrum/). To determine the relationship between photo counts and viral loads, a total of 84 nasopharyngeal swabs were collected from 17 patients at Kobe City Medical Center General Hospital and 67 commercially available samples (Cantor Bioconnect, Santee, CA, USA; validation dataset). For clinical performance analysis, a total of 115 nasopharyngeal swabs were collected from 115 patients at Juntendo University Hospital (test dataset).

### SARS-CoV-2 RT-qPCR

Viral RNAs were extracted using QIAamp Viral RNA Mini Kit (Qiagen, Hilden, Germany). RT-qPCR was performed following the protocol developed by the National Institute of Infectious Diseases of Japan. The primer/probe set (N2) was designed based on the N sequence of SARS-CoV-2 RNA (NC_045512.2)^26^. The primer/probe sequences were as following: forward primer (5′-AAATTTTGGGGACCAGGAAC-3′), reverse primer (5′-TGGCAGCTGTGTAGGTCAAC-3′), and TaqMan probe (5′-FAM-ATGTCGCGCATTGGCATGGA-BHQ-3′). The expected amplicon size was 158 bp. QuantiTect Probe RT-PCR Kit (Qiagen, Germantown, MD, USA) and Applied Biosystems 7500 Fast Real-Time PCR System (Thermo Fisher Scientific, Waltham, MA, USA) were used. The Ct values were assessed using the protocol developed by the National Institute of Infectious Diseases (version 2.9.1)^26^. The association between absolute viral copy numbers (viral loads) and Ct values was determined using SARS-CoV-2 Positive Control RNA (JP-NN2-PC**,** Nihon Gene Research Laboratories, Miyagi, Japan).

### Recombinant antigen production

For antigen cross-reactivity tests, nucleocapsid antigens derived from the other coronaviruses and influenza viruses were prepared. The recombinant nucleocapsid proteins were produced based on the following sequences: SARS-CoV (YP_009825061), Middle East respiratory syndrome-related coronavirus (MERS-CoV; YP_009047211), human coronavirus HKU1 HCoV-HKU1 (YP_173242), HCoV-OC43 (YP_009555245), HCoV-NL63 (YP_003771), and HCoV-229E (NP_073556). Each recombinant protein was prepared according to previously described methods^24^.

### Western blot

The detection of SARS-CoV-2 Antigens by SARS-CoV antibodies was confirmed via western blot analysis using the previously produced anti-N capsid SARS-CoV antibodies^24^. Histidine (His)-tagged SARS-CoV-2N proteins (10 ng) were loaded on NEXT Page II gel (5–20% gradient; Gellex, Tokyo, Japan), and electrophoresis was performed using iBind Western Device (Thermo Fisher Scientific). Two lots each of anti-SARS-CoV antibodies (0.5 μg/mL) and horseradish peroxidase (HRP)-conjugated anti-His antibodies (Penta-His Antibody; Cat. No. 34660, Qiagen) were used as primary antibodies. Anti-IgG antibodies (Medical & Biological Laboratories, Nagoya, Japan) were used as the secondary antibodies. Immobilon Western HRP substrate (Millipore, Burlington, MA, USA) was used for chemiluminescent detection.

### HISCL SARS-CoV-2 Antigen assay kit

Previously produced monoclonal antibodies against the SARS-CoV N proteins^24^ were used to probe the SARS-CoV-2N proteins (ACRO Biosystems, Newark, DE, USA). The HISCL SARS-CoV-2 Antigen assay kit is a chemiluminescent enzyme immunoassay that uses a HISCL automatic immunoassay analyzer (Sysmex, Kobe, Japan).

**Figure 5:**
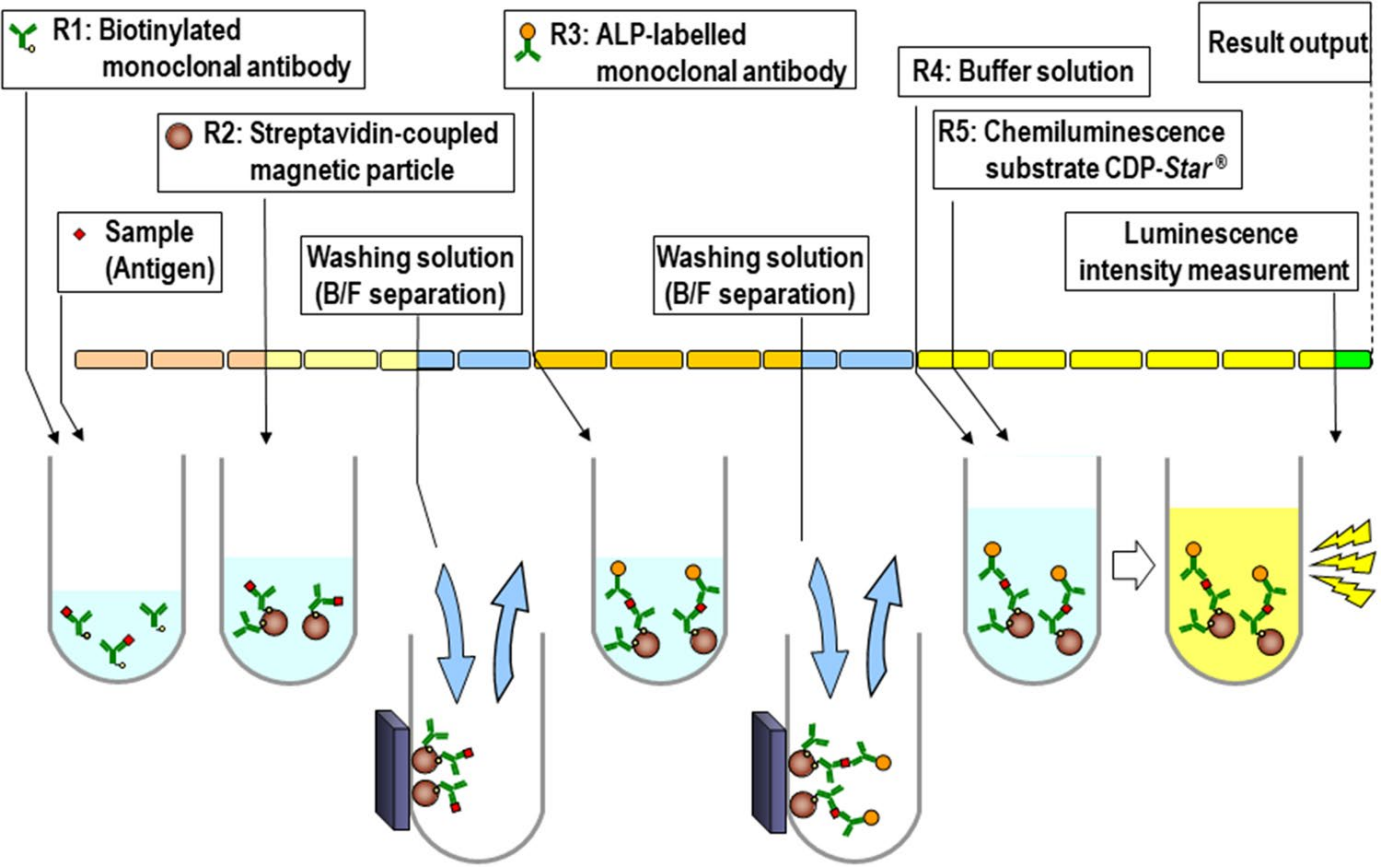
Assay protocol of the HISCL SARS-CoV-2 Antigen assay kit. The biotinylated SARS-CoV-2 Ag antibody (R1) was allowed to react with the sample at 42 °C for 3 min. Then, streptavidin-bonded magnetic particles (R2) were added and allowed to react at 42 ℃ for 2 min. After protein separation and washing, ALP-bound SARS-CoV-2 Ag antibody (R3) was added and reacted at 42 °C for 3 min. After another magnetic separation and washing, buffer solution (R4) and the chemiluminescent substrate (R5) were allowed to react at 42 °C for 5.5 min, then the luminescence intensity was measured. SARS-CoV-2: severe acute respiratory syndrome coronavirus 2; ALP: alkaline phosphatase; Ag: antigen. The illustration was drawn by A.K. using Microsoft PowerPoint 17.0 (Microsoft Corporation, Redmond, Washington: https://www.microsoft.com/en-us/microsoft-365/powerpoint).

Figure 5 presents a schematic illustration of the procedure: (1) the samples were incubated with biotinylated SARS-CoV-2 antibodies (R1: antibody 2–3) at 42 °C for 3 min; (2) the mixtures were incubated with streptavidin-bonded magnetic particles at 42 °C for 2 min; (3) after protein separation and washing, alkaline phosphatase (ALP)-bound SARS-CoV-2 antibodies (R3: antibody 2–12) were added and the mixtures were incubated at 42 °C for 3 min; (4) after magnetic separation and washing again, the chemiluminescent substrates were added and the mixtures were incubated at 42 °C for 5.5 min; and (5) chemiluminescence signals (CDP-Star, C0712, Sigma-Aldrich, St. Louis, MO, USA) were measured using the photo counter of HISCL-800 (Sysmex, Kobe, Japan). The level of SARS-CoV-2 Ag was indicated as cut-off index (COI), calculated by the difference in the luminescence intensities in the buffers with and without the SARS-CoV-2 antigens.

### Human sample collection

Human samples were obtained using nasopharyngeal cotton swabs following the standard method^36^. The swabs were immersed in 0.5 mL phosphate-buffered saline or viral transfer medium (Lampire Biological Laboratories, Pipersville, PA, USA). The suspensions were frozen at − 80 °C until antigen tests were performed. Highly viscous samples were centrifuged (2000×*g* for 5 min), and the supernatants were used for subsequent analyses.

### Reproducibility

Within-run and between-run reproducibility were determined by running the buffers with and without SARS-CoV-2 antigens. The recombinant human antigen for SARS-CoV-2 was purchased from ACRO Biosystems (Newark, DE). To test reproducibility, buffers containing SARS-CoV-2 antigens (positive control) and those without antigens (negative control) were prepared. To test between-run reproducibility, both negative and positive controls were tested twice per day each for five consecutive days.

### Cross-reaction

To determine specificity, cross-reactions were checked using measuring buffers containing various recombinant viral antigens: human SARS-CoV, MERS-CoV, HCoV-229E, HCoV-OC43, HCoV-NL63, HCoV-HKU1, influenza virus H1N1, influenza virus H3N2, and influenza virus B. Inactivated influenza viruses were purchased from Advanced Biotechnologies (Eldersburg, MD, USA). The antigens of SARS-CoV, MERS-CoV, HCoV-229E, HCoV-OC43, HCoV-NL63, and HCoV-HKU1 were prepared according to a previous established protocol^24^. HISCL SARS-CoV-2 antigen assays were carried out following the manufacturer’s instructions.

### Statistical analyses

Statistical analyses were performed in Excel (Microsoft, Redmond, WA, USA). Receiver operating characteristic (ROC) curve analyses were conducted using GraphPad Prism 9.0.0 (GraphPad Software: https://www.graphpad.com/support/faq/prism-900-release-notes/, San Diego, USA) to evaluate the assay performance and visualize the curves. The areas under the ROC curves (AUCs), sensitivity, and specificity were calculated.

## Supplementary Information

Supplementary Information.

## Data Availability

The datasets supporting the conclusions of this article is(are) included within the article (and its additional files).
