# Supplementary material for: Performance and usefulness of a novel automated immunoassay HISCL SARS-CoV-2 Antigen assay kit for the diagnosis of COVID-19
Source: Sci Rep. 2021 Dec 1;11:23196. doi: 10.1038/s41598-021-02636-x (PMC8636628; doi:10.1038/s41598-021-02636-x)
Supplement: Supplementary file 1 — Supplementary Information. [file 41598_2021_2636_MOESM1_ESM.pdf]

Supplemental materials

Performance and usefulness of a novel automated immunoassay HISCL SARS-CoV-2 Antigen assay kit for the diagnosis of COVID-19

Kaori Saito[1†], Tomohiko Ai[1†], Akinori Kawai[2], Jun Matsui[3], Yoshiyuki Fukushima[3], Norihiro Kikukawa[2], Takuya Kyoutou[4], Masayoshi Chonan[5], Takeaki Kawakami[5], Yoshie Hosaka[5], Shigeki Misawa[5], Haruhi Takagi[6], Yasushi Matsushita[7], Makoto Hiki[8,9], Atsushi Okuzawa[10], Satoshi Hori[6,11], Toshio Naito[12], Takashi Miida[1], Kazuhisa Takahashi[6], Yoko Tabe[1,13*]

[1]Department of Clinical Laboratory Medicine, Juntendo University Graduate School of Medicine, Tokyo, Japan

[2]Clinical Innovation, Sysmex Corporation, Kobe, Japan

[3]LS Medical Affairs, Sysmex Corporation, Kobe, Japan

[4]Engineering 1, Sysmex Corporation, Kobe, Japan

[5]Department of Clinical Laboratory, Juntendo University Hospital, Tokyo, Japan

[6]Department of Respiratory Medicine, Juntendo University Graduate School of Medicine, Tokyo, Japan

[7]Department of Internal Medicine and Rheumatology, Juntendo University Graduate School of Medicine, Tokyo, Japan

[8]Emergency and Disaster Medicine, Juntendo University Faculty of Medicine, Tokyo, Japan

[9]Department of Cardiovascular Biology and Medicine, Juntendo University Faculty of Medicine, Tokyo, Japan

[10]Department of Infection Control Science, Juntendo University Graduate School of Medicine, Tokyo, Japan

[11]Department of Coloproctological Surgery, Juntendo University Graduate School of Medicine, Tokyo, Japan

[12]Department of General Medicine, Juntendo University Graduate School of Medicine, Tokyo, Japan

[13]Department of Next Generation Hematology Laboratory Medicine, Juntendo University Graduate School of Medicine, Tokyo, Japan

[†]These authors equally contributed to the study.

Supplemental Table 1. Validation dataset

	HICL			RT-PCR
	Count	Ag (pg/mL)	COI	Copy/test
1	9124	0.7	0.2	17
2	7431	<0.00	0.0	20
3	13287	4.7	1.3	21
4	7718	<0.00	0.0	26
5	11041	2.5	0.7	48
6	13905	5.3	1.5	56
7	12794	4.2	1.2	63
8	12709	4.2	1.1	65
9	13905	5.3	1.5	80
10	10782	2.3	0.6	81
11	10073	1.6	0.4	292
12	314223	295.8	81.1	1087
13	254478	238.0	65.2	1499
14	485100	461.1	126.3	1998
15	151518	138.4	37.9	2077

16	92006	80.9	22.2	4367
17	102314	90.8	24.9	13196
18	433187	410.9	112.6	13291
19	2715600	2618.9	717.5	27682
20	195055	180.6	49.5	47208
21	380159	359.6	98.5	81391
22	803453	769.1	210.7	115743
23	2796673	2697.3	739.0	618395
24	35994811	34812.4	9537.6	1121785
25	17744166	17157.1	4700.6	1246365
26	16745726	16191.3	4436.0	1680481
27	40782409	39443.8	10806.5	4369413
28	97282701	94100.8	25781.1	8277600
29	155335619	150259.8	41167.1	15067576
30	153011627	148011.7	40551.1	45134920
31	8651	0.2	0.1	0
32	8065	0.0	0.0	0
33	8167	0.0	0.0	0
34	7825	0.0	0.0	0
35	8110	0.0	0.0	0
36	8221	0.0	0.0	0
37	7286	0.0	0.0	0
38	6632	0.0	0.0	0
39	7773	0.6	0.2	0
40	8632	1.5	0.4	0
41	6816	0.0	0.0	0
42	4662	0.0	0.0	0
43	9998	3.1	0.8	0

44	9761	2.8	0.8	0
45	9657	2.7	0.7	0
46	9714	2.8	0.8	0
47	9502	2.5	0.7	0
48	9423	2.4	0.7	0
49	7238	0.0	0.0	0
50	8864	1.8	0.5	0
51	7866	0.7	0.2	0
52	9122	2.1	0.6	0
53	13174	6.7	1.8	0
54	6715	0.0	0.0	0
55	8749	1.6	0.4	0
56	8820	1.7	0.5	0
57	8138	0.9	0.2	0
58	8782	1.6	0.4	0
59	8346	1.1	0.3	0
60	8781	1.6	0.4	0
61	8506	1.3	0.4	0
62	8588	1.4	0.4	0
63	8666	1.5	0.4	0
64	8490	1.3	0.4	0
65	8075	0.8	0.2	0
66	8506	1.3	0.4	0
67	7785	0.5	0.1	0
68	8209	1.0	0.3	0
69	8555	1.4	0.4	0
70	7975	0.7	0.2	0
71	8625	1.5	0.4	0

72	8221	1.0	0.3	0
73	8570	1.4	0.4	0
74	8427	1.2	0.3	0
75	8011	0.8	0.2	0
76	8758	1.6	0.4	0
77	8298	1.1	0.3	0
78	8546	1.4	0.4	0
79	8287	1.1	0.3	0
80	8308	1.1	0.3	0
81	7898	0.6	0.2	0
82	7771	0.5	0.1	0
83	8727	1.6	0.4	0
84	8606	1.4	0.4	0

Supplemental Table 2. Test Dataset

ID	Copy numbers	Ct	COI	Day	Severity
Cov-00102	133789	23.43	398.8	0	mild
Cov-00120	10	36.22	39.8	0	mild
Cov-03394	12025642	17.78	38233.6	1	mild
Cov-05261	1760925	20.6	29609.9	1	mild
Cov-03406	19019181	16.3	5763.6	2	mild
Cov-00043	158651	23.15	998.2	3	mild
Cov-00041	79628	22.82	1617.3	4	mild
Cov-00134	922	30.49	13.4	4	mild
Cov-00061	789139	20.59	3211.2	5	mild
Cov-04117	8799	28	262.2	5	mild
Cov-00035	469	31.5	112.9	5	mild
Cov-00059	305	31.11	22.8	5	mild
Cov-00039	49	33.87	<1.0	5	mild
Cov-00034	1060	29.23	29.2	6	mild
Cov-00098	7	37.9	8.9	6	mild

Cov-00068	2	39.41	<1.0	6	mild
Cov-04049	258	32.41	<1.0	8	mild
Cov-00249	2300950	19.31	6449.6	9	mild
Cov-00062	2	38.68	18.6	9	mild
Cov-00931	4	37.74	<1.0	17	mild
Cov-00463	3	38.25	<1.0	18	mild
Cov-00428	2	38.84	<1.0	21	mild
Cov-00513	22	35.06	<1.0	22	mild
Cov-00462	2	38.57	<1.0	22	mild
Cov-00442	2	38.89	<1.0	22	mild
Cov-00402	17	35.52	<1.0	25	mild
Cov-03461	4	37.89	<1.0	25	mild
Cov-04948	1	39.97	<1.0	25	mild
Cov-04946	14	36.76	<1.0	27	mild
Cov-00545	2	38.57	<1.0	35	mild
Cov-03196	605040	21.74	1329.1	N/A	mild
Cov-03889	58992	24.23	797.5	N/A	mild
Cov-02364	75	34.26	<1.0	N/A	mild
Cov-00205	1	40.34	<1.0	N/A	mild
Cov-00073	812321	20.4	8056	0	moderate
Cov-00183	423	30.62	2.8	0	severe
Cov-00148	226409	22.74	1241.3	4	moderate
Cov-00091	140	32.29	5.5	5	critical
Cov-00227	118554	22.83	233.9	7	moderate
Cov-00733	20	35.26	<1.0	8	moderate
Cov-00142	2583175	19.02	6328.7	13	moderate
Cov-00404	52	33.78	<1.0	14	moderate
Cov-00497	1	40.03	2.6	20	severe
Cov-00443	2	39.15	<1.0	21	moderate
Cov-00364	1	39.74	<1.0	23	moderate
Cov-00444	2	38.78	<1.0	28	moderate
Cov-00514	-	-	<1.0	33	
Cov-00515	-	-	<1.0	26	
Cov-00506	-	-	<1.0	0	
Cov-00308	-	-	<1.0	N/A	
Cov-00481	-	-	<1.0	30	
Cov-00479	-	-	<1.0	23	
Cov-00535	-	-	<1.0	28	
Cov-00406	-	-	<1.0	19	
Cov-00492	-	-	<1.0	23	
Cov-01441	-	-	<1.0	2	

Cov-01433	-	-	<1.0	N/A	
Cov-01448	-	-	<1.0	N/A	
Cov-01419	-	-	<1.0	N/A	
Cov-01447	-	-	<1.0	N/A	
Cov-01434	-	-	<1.0	N/A	
Cov-01417	-	-	<1.0	N/A	
Cov-01444	-	-	<1.0	N/A	
Cov-01460	-	-	<1.0	1	
Cov-01456	-	-	<1.0	N/A	
Cov-01464	-	-	<1.0	N/A	
Cov-01416	-	-	<1.0	N/A	
Cov-01424	-	-	<1.0	N/A	
Cov-01453	-	-	<1.0	N/A	
Cov-01466	-	-	<1.0	N/A	
Cov-01463	-	-	<1.0	4	
Cov-01467	-	-	<1.0	9	
Cov-01446	-	-	<1.0	N/A	
Cov-01455	-	-	<1.0	N/A	
Cov-01445	-	-	<1.0	3	
Cov-01421	-	-	<1.0	N/A	
Cov-01439	-	-	<1.0	1	
Cov-01450	-	-	<1.0	N/A	
Cov-01411	-	-	<1.0	N/A	
Cov-01458	-	-	<1.0	N/A	
Cov-01408	-	-	<1.0	N/A	
Cov-01461	-	-	<1.0	5	
Cov-01429	-	-	<1.0	N/A	
Cov-01462	-	-	<1.0	1	
Cov-01425	-	-	<1.0	N/A	
Cov-01437	-	-	<1.0	2	
Cov-01418	-	-	<1.0	N/A	
Cov-01430	-	-	<1.0	N/A	
Cov-01435	-	-	<1.0	1	
Cov-01409	-	-	<1.0	0	
Cov-01412	-	-	<1.0	2	
Cov-01459	-	-	<1.0	N/A	
Cov-01420	-	-	<1.0	N/A	
Cov-01427	-	-	<1.0	N/A	
Cov-01443	-	-	<1.0	1	
Cov-01454	-	-	<1.0	N/A	
Cov-01426	-	-	<1.0	N/A	

Cov-01451	-	-	<1.0	N/A	
Cov-01413	-	-	<1.0	6	
Cov-01423	-	-	<1.0	N/A	
Cov-01410	-	-	<1.0	N/A	
Cov-01414	-	-	<1.0	3	
Cov-01428	-	-	<1.0	N/A	
Cov-01431	-	-	<1.0	N/A	
Cov-01422	-	-	<1.0	N/A	
Cov-01432	-	-	<1.0	N/A	
Cov-01415	-	-	<1.0	0	
Cov-01449	-	-	<1.0	N/A	
Cov-01438	-	-	<1.0	N/A	
Cov-01436	-	-	<1.0	N/A	
Cov-01440	-	-	<1.0	2	
Cov-01465	-	-	<1.0	0	
Cov-01457	-	-	<1.0	N/A	
Cov-01452	-	-	<1.0	N/A	
Cov-01442	-	-	<1.0	0	

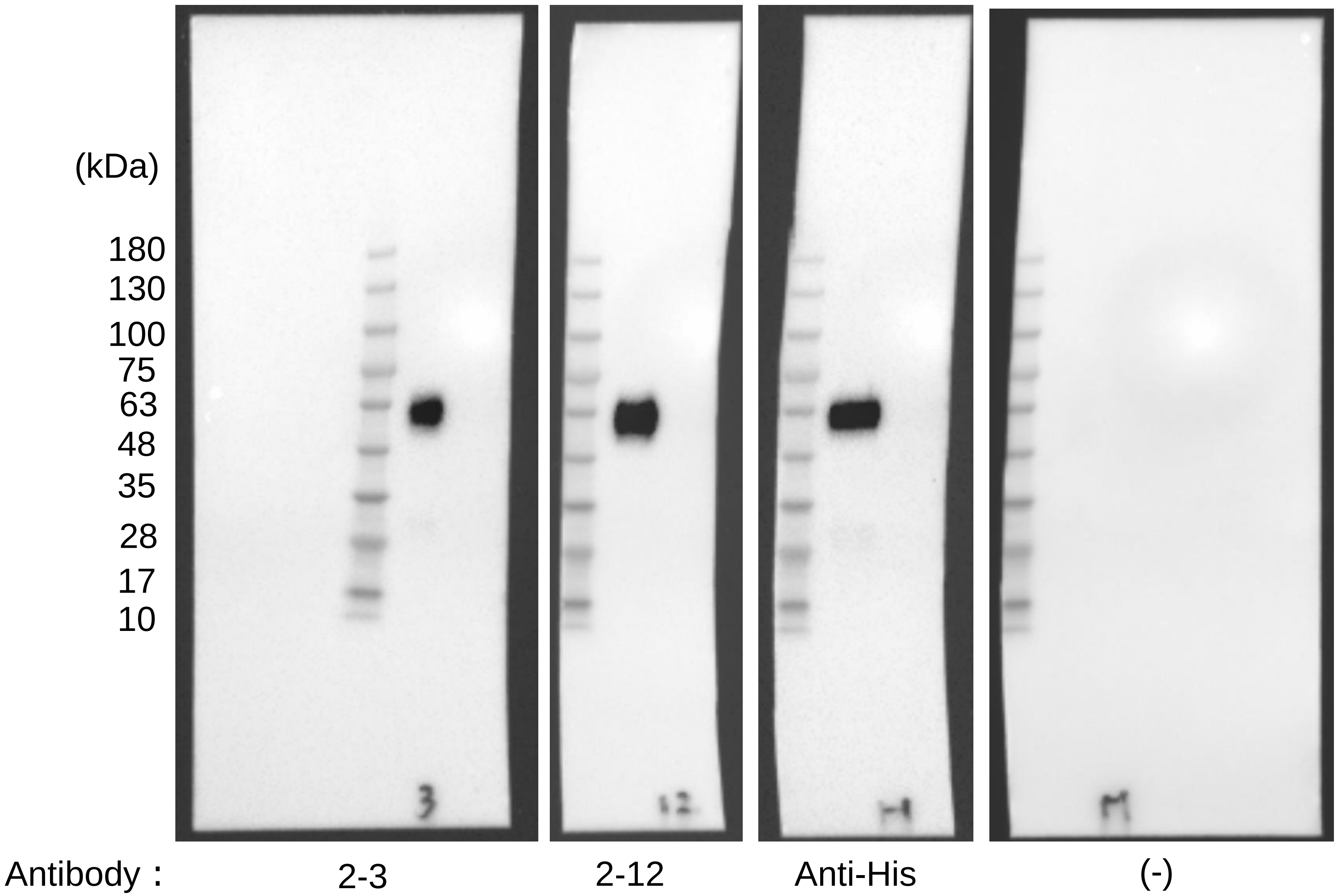

Supplemental Figure 1. The original picture of the western blot results before crop.
